# Legionnaires' Disease with Facial Nerve Palsy

**DOI:** 10.1155/2011/916859

**Published:** 2011-03-10

**Authors:** Shailesh R. Basani, Salwa Mohamed Ahmed, Eyassu Habte-Gabr

**Affiliations:** Department of Internal Medicine, Hurley Medical Center, Flint, MI 48503, USA

## Abstract

Legionnaires' disease is primarily a pneumonic process caused by *Legionella pneumophilia*, a gram-negative aerobic bacillus but also has multiple system involvement. The most common manifestation is encephalopathy suggesting a generalized brain dysfunction but focal neurological manifestations have been reported. We report a patient with Legionella pneumonia associated with cerebellar dysfunction and unilateral facial nerve weakness. 51-year-old previously healthy male presented with shortness of breath, cough, slurred speech, and unsteadiness on feet associated with malaise, fevers and myalgias. Patient's family reported facial asymmetry for 2 days. Patient had no significant medical history and was not on any medication. He denied smoking, alcohol or illicit drug use. Chest X-ray showed bilateral lower lobe infiltrates. Urinary antigen assay for *Legionella pneumophilia* serogroup 1 was positive. Patient was started on intravenous moxifloxacin. On day 5 the patient was discharged home and continued oral moxifloxacin for two weeks. After the two weeks, his respiratory symptoms, gait ataxia and dysarthria resolved. We report the first case of Legionnaires' disease with cerebellar dysfunction and seventh nerve palsy. Legionnaires' disease should be considered in patients with any neurological symptoms in the setting of pneumonia. Failure to recognize and treat the infection may lead to poor outcomes.

## 1. Introduction

Legionnaires' disease was described initially in 1976 among delegates of American Legion Convention in Philadelphia [1]. It is primarily a pneumonic process caused by Legionella pneumophilia, a gram-negative aerobic bacillus but also has multiple system involvement. Neurological manifestations are well recognized and may develop in 40%–50% of patients [2]. The most common manifestation is encephalopathy suggesting a generalized brain dysfunction but focal neurological manifestations such as brainstem dysfunction, cranial nerve dysfunction, and peripheral neuropathy have also been reported as rare occurrence [3, 4]. Reports of isolated cranial nerve involvement were of 3rd nerve, 6th nerve, and 7th nerve dysfunction. This paper describes a patient with Legionella pneumonia associated with cerebellar dysfunction and unilateral facial nerve weakness.

## 2. Case Report

A 51-year-old previously healthy male presented to our department with complaints of shortness of breath, cough, slurred speech, unsteadiness on feet associated with malaise, fevers, and myalgias. The symptoms began seven days prior to presentation with progressively worsening fevers with chills, generalized myalgias, and malaise. Two days later, he developed dyspnea and dry cough. He also complained of nausea, vomiting with watery diarrhea, headache with slurred speech, and feeling unsteady on the feet. The patient's family reported facial asymmetry for last 2 days. 

Patient denied any hemoptysis, head trauma, loss of consciousness, dizziness, or confusion. He worked as a security guard in a local company. He denied recent travel or any contact with sick patients. Patient did not have any significant past medical history and is not on any medication. He denied smoking, alcohol use, or any illicit drug usage.

On physical examination, he appeared ill, diaphoretic, and in respiratory distress. His vital signs were as follows: respiratory rate was 24 per min, pulse rate 102 per min, blood pressure 140/73 mm of Hg, SpO_2_ of 95% on room air, and oral temperature 39.3°C. His chest examination revealed inspiratory crackles in both the lower lung fields. Neurological examination revealed alert and oriented man with dysarthria, but tongue movement was normal and in midline. He had no nystagmus or gaze paralysis. He had upper motor neuron-type weakness of left facial nerve. Gait was ataxic with positive Romberg's test. He had normal tone, strength, reflexes, and coordination in all the four extremities. There was no neck rigidity.

**Figure 1:**
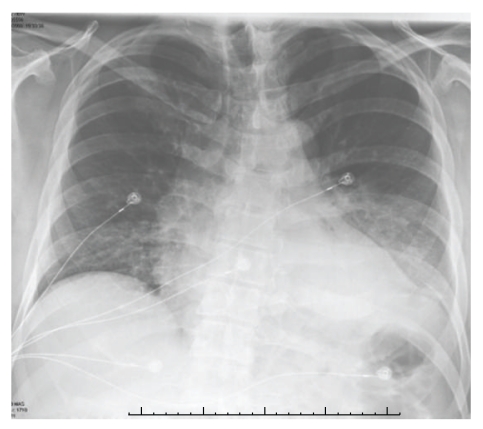
Chest X-ray on day 1 showing bilateral basal infiltration.

Laboratory abnormalities are as shown in Table 1. Chest X-ray (shown in Figure 1) showed bilateral lower lobe infiltrates. EKG showed sinus tachycardia at rate of 102/min but no other acute changes. Bacterial blood cultures were negative. Urinary antigen assay for Legionella pneumophilia serogroup 1 was positive. Sputum cultures were negative for Legionella. Noncontrast CT of brain was normal. Magnetic resonance imaging (MRI) scan of the brain with gadolinium was normal. Electroencephalography (EEG) showed generalized slowing of the background activity suggestive of mild encephalopathy but no focal spikes.

**Figure 2:**
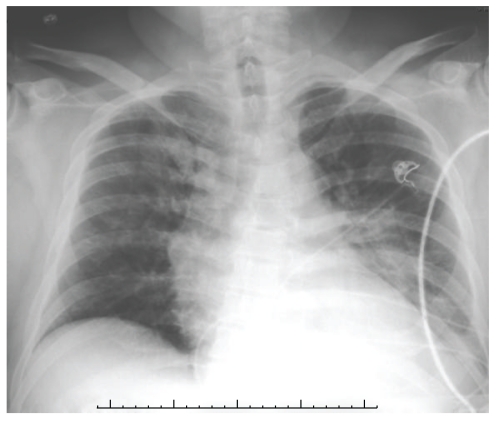
Chest X-ray on day 4 showing resolution of the earlier changes.

The patient was started on intravenous moxifloxacin along with the routine supportive care. By day 4, diarrhea completely resolved. His respiratory symptoms and dysarthria also started improving, but the ataxia and facial weakness persisted. His chest X-ray (shown in Figure 2) at this time was clear of any infiltration that was noted on admission. 

On day 5, the patient was discharged home with plan of continuing oral moxifloxacin for two more weeks. Neurological examination at this time revealed mild dysarthria, mild ataxia needing a walking aid, and persisting facial weakness. 

He was followed up in our outpatient clinic in 2 weeks. At this point, his respiratory symptoms, gait ataxia, and dysarthria completely resolved. His facial weakness improved moderately, but there was still some facial asymmetry. A chest X-ray repeated at this time was normal.

At 3 months, the patient still had persisting unilateral facial weakness.

## 3. Discussion

Legionnaires' disease was described in 1976 in delegates of American Legion Convention [1]. It is now described as an infection with multisystem involvement [5]. Neurological complications occur in 40%–50% of cases [2]. Common neurological manifestations include headache, somnolence, and varying degrees of encephalopathy. Less frequent presentations are brain stem dysfunction, cerebellar dysfunction, peripheral neuropathies, pyramidal tract dysfunction, cranial nerve palsies, memory loss, seizures, affective disorders, and hallucinations [3, 4].

The cranial nerve palsies that have been described so far are occulomotor and abducens nerve involvement and facial nerve [4, 6, 7]. 

Our patient presented with symptoms consistent with pneumonia along with focal neurological signs. The diagnosis of legionella pneumonia was made by detection of *L. pneumophila* serogroup 1 antigenuria by immune assay. Brain CT, MRI, and EEG were normal. Thyroid function tests were normal. Antinuclear antibody screen and C3 and C4 complement levels were normal to exclude any vasculitic process. As the neurological symptoms and pneumonia occurred concurrently and the improvement in ataxia, dysarthria, and facial weakness occurred with the treatment of pneumonia, we presumed that the neurological symptoms were due to Legionella pneumonia. 

A computer-based search of literature (MEDLINE; years 1948–December 2010) without language restriction was performed. Key words used in the search were *legionella, legionella pneumophila, legionnaires' disease, legionellosis, cranial nerves, nerve, cerebellar, and neurology. *The citations in all identified articles were evaluated and English abstracts of foreign language articles were also evaluated. The combination of Legionnaires' disease and seventh nerve involvement has never been reported in the past [4, 8].

We report the first case of Legionnaires' disease with cerebellar dysfunction and seventh nerve palsy. The cerebellar involvement was mild affecting the trunk presenting as gait ataxia and dysarthria. There was no nystagmus or lateralizing signs. The seventh nerve palsy was unilateral and upper motor neuron type. 

Cerebellar dysfunction is rare but well documented in Legionnaires' disease. Shelburne et al. reported 29 cases of cerebellar dysfunction in Legionnaires' disease [9], and Johnson et al. reported the incidence of cerebellar dysfunction is 3.7% in Legionnaires' disease [2]. As per Shelburne et al. the most frequently reported cerebellar symptoms in Legionnaires' disease are dysarthria (79%) and ataxia (72%) [9].

In literature, we came across only two cases with facial nerve palsy in the setting of legionellosis, and they were different from our case for multiple reasons.

The case of bilateral facial nerve palsy reported by Morgan and Gawler was in the setting of severe and progressive peripheral neuropathy and encephalopathy [8] which was not present in our patient. The type of facial weakness has not been clearly described. The facial nerve palsy in our patient was upper motor neuron type as per our clinical examination.

The second case was reported by Johnson et al., in the setting pneumonia and confusion on admission, rapidly progressed to coma and later developed left facial nerve palsy and hemiparesis. Nuclide brain scan with technetium −99-m pertechnate which was normal on admission, later, it demonstrated multiple focal defects in both the hemispheres. The patient eventually succumbed to severe sepsis, and at autopsy, necrotizing hemorrhagic leucoencephalitis with bacilli morphologically similar to legionella has been identified. In this case, secondary causes for the neurological presentations such as a vascular event could have been possible [2].

The pathogenesis of neurological dysfunction in Legionnaires' disease is unclear. Various theories have been proposed suggesting direct invasion, toxin, or immune-mediated or reversible ischemia. Legionella has been identified in brain on rare instances in autopsy reports suggesting direct invasion of central nervous system [4, 10, 11]. In the absence of direct invasion, neurotoxin, or immune-mediated mechanisms have been proposed as possible mechanisms of neurological involvement. However, clear evidence supporting either of the mechanisms is lacking [12]. 

Focal areas of ischemia has been suggested by Imai et al. by demonstrating multiple areas of hypoperfusion in cerebellum and frontal lobe using single photon emission computed tomography (SPECT) with Tc-99 m hexamethyl-propyleneamine oxime (HMPAO) reconstructed using the three-dimensional stereotactic surface projection (3D-SSP) technique 3D [13]. Their patient had a reversible lesion in the splenium of corpus callosum, identified on magnetic resonance imaging (MRI) scan of the brain.

The utility of functional imaging of brain such as single photon emission computed tomography (SPECT) and positron emission tomography (PET) in diagnosis of Legionnaires' disease with neurological dysfunction was predicted by Morgan et al. [14].

The treatment of this condition is antibiotics and supportive care. High-dose corticosteroids have been used in life-threatening conditions, and the evidence for it is only anecdotal [12]. 

The prognosis of neurological deficits is not well studied due to the limited number of cases. In general, the encephalopathy and coma are largely reversible with mild residual deficits. The focal deficits such as cerebellar and gait disturbance tend to persist for long periods [4, 12].

In retrospect, we identified the following investigations which would have been interesting in our patient. Although the patient management and outcome would not have changed, functional brain imaging could have helped us to localize the lesion. Though the utility of cerebrospinal fluid analysis (CSF) is undetermined, it may be helpful to rule out other infective causes.

In conclusion, Legionnaires' disease should be strongly considered in patients with any neurological symptoms in the setting of pneumonia. In such a setting, full evaluation has to be performed to exclude other etiologies. Failure to recognize and treat the infection may lead to poor outcomes.

## Figures and Tables

**Table 1:** Abnormal laboratory data.

Sodium	130 meq/L
potassium	3.3 meq/L
BUN	25 mg/dL
creatinine	1.1 mg/dL
alanine aminotransferase	231 U/L
aspartate aminotransferase	378 U/L
myoglobin	665 ng/mL
creatinine phosphokinase	721 U/L
C-reactive protein	9.2 mg/dL

## References

[1] Fraser DW, Tsai TR, Orenstein W (1977). Legionnaires’ disease: description of an epidemic of pneumonia. *The New England Journal of Medicine*.

[2] Johnson JD, Raff MJ, Van Arsdall JA (1984). Neurologic manifestations of Legionnaires’ disease. *Medicine*.

[3] Plaschke M, Strohle A, Then Bergh F, Backmund H, Trenkwalder C (1997). Neurologic and psychiatric symptoms of legionella infection. Case report and overview of the clinical spectrum. *Nervenarzt*.

[4] Johnson JD, Raff MJ, Van Arsdall JA (1984). Neurologic manifestations of Legionnaires’ disease. *Medicine*.

[5] Stout J, Yu V (1997). Legionellosis. *The New England Journal of Medicine*.

[6] Reynaga E, Pedro-Botet ML, Lozano-Sánchez M, Sopena N, Sabriá M (2001). Third cranial nerve palsy and Legionnaire’s disease. *Revista de Neurologia*.

[7] Lattimer GL, Rhodes LV (1978). Legionnaires’ disease. Clinical findings and one-year follow-up. *Journal of the American Medical Association*.

[8] Morgan DJR, Gawler J (1981). Severe peripheral neuropathy complicating legionnaires’ disease. *British Medical Journal*.

[9] Shelburne SA, Kielhofner MA, Tiwari PS (2004). Cerebellar involvement in legionellosis. *Southern Medical Journal*.

[10] Andersen BB, Sogaard I (1987). Legionnaires’ disease and brain abscess. *Neurology*.

[11] Schurmann D, Grosse G, Horbach I, Fehrenbach FJ (1983). Pulmonary and extrapulmonary manifestations of L. pneumophila. fur bakteriologie, mikrobiologie und hygiene - 1 - abt - originale A, medizinische mikrobiologie. *Infektionskrankheiten und Parasitologie*.

[12] Kulkarni KH, Thorat SB, Wagle SC, Khadilkar SV (2005). Focal neurological manifestations in legionellosis. *Journal of Association of Physicians of India*.

[13] Imai N, Yagi N, Konishi T, Serizawa M, Kobari M (2008). Legionnaires’ disease with hypoperfusion in the cerebellum and frontal lobe on single photon emission computed tomography. *Internal Medicine*.

[14] Morgan JC, Cavaliere R, Juel VC (2004). Reversible corpus callosum lesion in legionnaires’ disease. *Journal of Neurology, Neurosurgery and Psychiatry*.

